# Meibomian gland dysfunction and dry eye in keratoconus

**DOI:** 10.5935/0004-2749.20220056

**Published:** 2025-08-21

**Authors:** Roberto Damian Pacheco Pinto, Ricardo Yuji Abe, Flávia Cid Gomes, Alexandre Fattah Martini, Eduardo Buzolin Barbosa, Monica Alves

**Affiliations:** 1 Discipline of Ophthalmology and Otorhinolaryngology, Faculdade de Ciências Médicas, Universidade Estadual de Campinas, Campinas, SP, Brazil; 2 Hospital Oftalmológico de Brasília, Brasília, DF, Brazil

**Keywords:** Keratoconus, Meibomian gland dysfunction, Blepharitis, Dry eye syndromes, Humans, Case reports, Ceratocone, Disfunção da glândula tarsal, Blefarite, Síndromes dos olho seco, Humanos, Relatos de casos

## Abstract

The objective of this report is to describe a case of meibomian gland dysfunction associated with keratoconus and to examine the importance of treatment for evaporative dry eye in cases of corneal ectasia. A 45-year-old man diagnosed as having keratoconus complained of burning, tearing, itching, and red eye. He had a history of penetrating corneal transplantation and wearing rigid contact lenses. The meibography revealed a severe meibomian gland dropout and normal tear meniscus height in both eyes. Objective propaedeutic tests are important tools for dry eye diagnosis and proper evaluation of ocular surface and tear film. In older patients, the classic signs of atopic conjunctivitis are not always present, and the causes of chronic rubbing must be further investigated. Treatment of underlying chronic inflammation such as dry eye, meibomian gland dysfunction, and blepharitis might be important to prevent keratoconus progression and guarantee symptom relief.

## Abstract (pt)

O objetivo é relatar um caso de associação entre disfunção de glândulas de meibomius (DGM) e ceratocone, assim como ressaltar a importância do tratamento do olho seco evaporativo em casos de ectasia corneana. Paciente do sexo masculino de 45 anos com ceratocone e queixas de ardência, lacrimejamento, prurido e olho vermelho. O mesmo tem histórico de transplante de córnea penetrante e uso de lentes de contato rígidas. A meibografia revelou severa perda de glândulas de meibomius e menisco lacrimal normal em ambos os olhos. Testes propedêuticos objetivos são importantes ferramentas para diagnóstico de olho seco e apropriada avaliação da superfície ocular e filme lacrimal. Em pacientes mais velhos, os sinais clássicos de atopia não estão sempre presentes e investigações adicionais das causas de prurido crônico são necessárias. O tratamento da inflamação crônica subjacente como olho seco, disfunção de glândulas de meibomius e blefarite podem ser importantes para prevenir a progressão do ceratocone e garantir alívio dos sintomas.

## INTRODUCTION

Keratoconus is a degenerative corneal disorder in which structural changes cause thinning and develop a more conical shape than the normal gradual curve. The exact cause of keratoconus is uncertain, but it has been associated with detrimental enzyme activity in the cornea. A genetic link seems likely, as the incidence rate is greater if keratoconus or atopic phenotypes have been diagnosed in a family member^(1)^.

Synthesis of the literature enabled the formulation a plausible hypothesis that epithelial microtrauma might lead to progression of keratoconus^(2)^. Therefore, measures to prevent ocular rubbing is a highly desirable measure to reduce epithelial trauma.

One of the most common causes of eye rubbing in the general population is chronic blepharitis. The common symptoms associated with blepharitis are burning sensation, irritation, tearing, photophobia, blurred vision, and red eye. Nevertheless, meibomian gland dysfunction (MGD)-associated posterior blepharitis is considered the main cause of dry eye disease, which leads to an evaporative subtype. The tear lipid layer is derived from the meibomian glands, which are of utmost importance for preserving the ocular surface to prevent tear evaporation. MGD-related dry eye can be diagnosed using direct methods such as meibography, which uses transillumination or infrared light to image the meibomian glands^(3)^. Meibography provides a feasible method of documenting and evaluating the morphology of the meibomian glands for better diagnosis of MGD and its severity in various related conditions.

In addition, a previous study showed that patients with keratoconus had increased prevalence rates of the signs and symptoms of blepharitis and dry eye syndrome as compared with healthy medical staff, who were included in the study as a control group^(4)^. Carracedo et al.^(5)^ reported that patients with keratoconus showed more severe symptoms of dry eye and tear instability, primarily due to the decreased mucin production, than healthy patients with no keratoconus. Chronic inflammation of the ocular surface, which is affected by the severity of blepharitis and other factors such as eye rubbing, may lead to the development of keratoconus and its progression. The possible mechanism is that microtrauma from chronic mechanical rubbing, a characteristic feature of chronic blepharitis, may be involved in the pathogenesis of keratoconus. Therefore, chronic inflammation and inflammatory mediators in patients with blepharitis may play a role in the etiology of keratoconus and its progression, as inflammatory mediators have been shown to contribute to the pathophysiology of keratoconus^(4)^. The fact that most cases of blepharitis do not progress to keratoconus can be explained by the multifactorial nature of the disease, which is yet to be well established.

The aim of this case report is to demonstrate the association between MGD and keratoconus and examine the importance of treatment for evaporative dry eye in cases of corneal ectasia.

## CASE REPORT

Male patients aged 45 years old who were diagnosed as having keratoconus at 20 years old and had a family history of keratoconus in his brother was evaluated at the Cornea and External Disease Unit of the Department of Ophthalmology and Otorhinolaryngology, University of Campinas. The patient denied other comorbidities or systemic medication use. He underwent penetrating corneal transplantation in the right eye 8 months before due to hydrops and had been wearing a rigid gas permeable (RGP) contact lens (CL) in the left eye since then. His visual acuity was 0.4 (decimal) in both eyes. He reported important symptoms of dryness and irritation and CL intolerance. He worked as a photographer, with frequent computer use.

**Figure 1:**
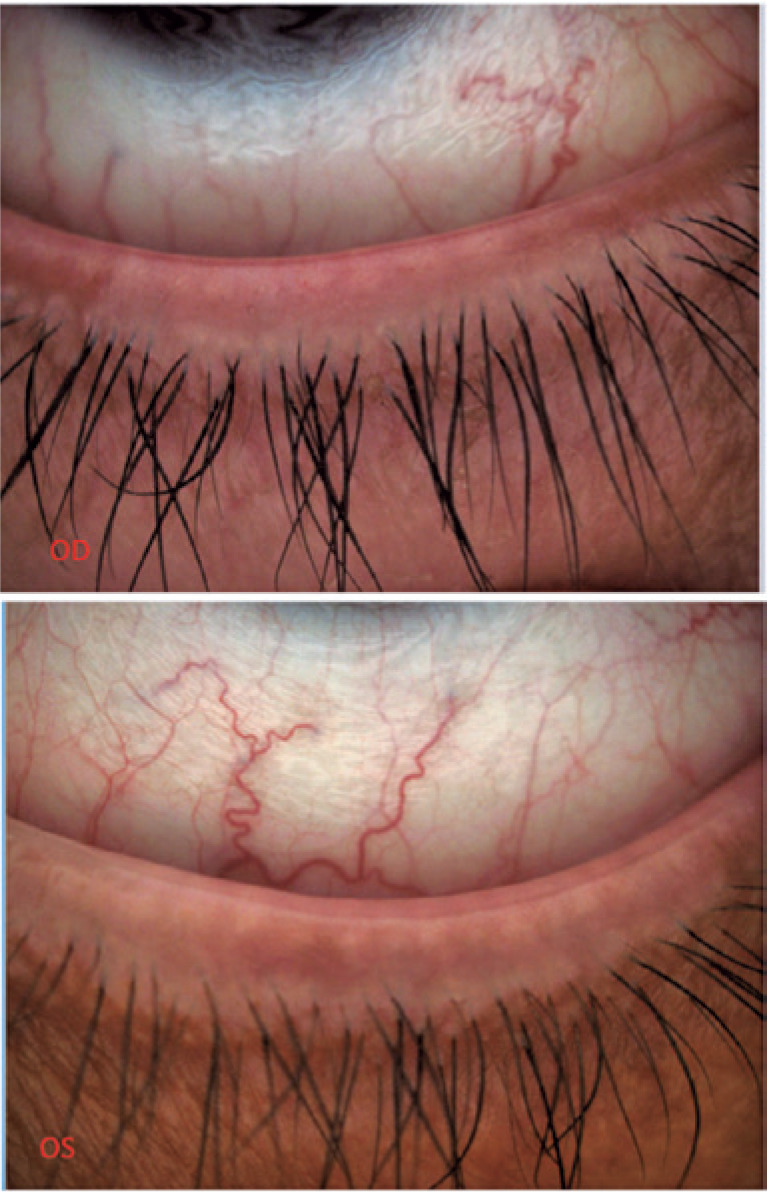
The eyelid showing mild anterior blepharitis and meibomian gland ductal obstruction.

Biomicroscopic evaluation revealed conjunctival hyperemia, eyelid margin erythema, and telangiectasia, and mild anterior blepharitis. The meibomian gland ducts were plugged, showing no release of oil secretions during eyelid expression (Figure 1).

OD: Right eye. OS: Left eye.

**Figure 2:**
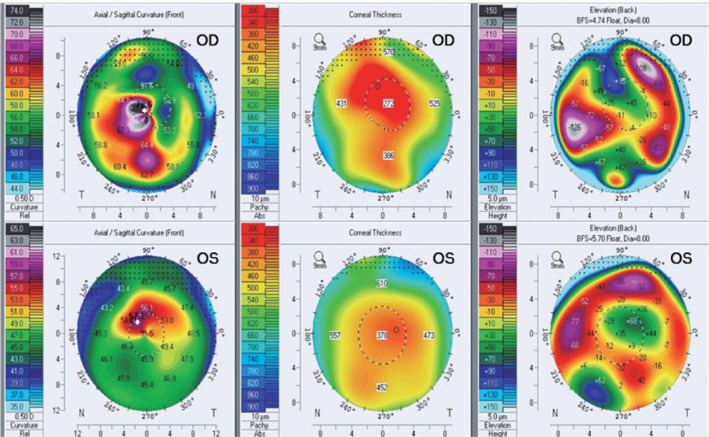
Corneal tomographic maps of both eyes.

Scheimpflug corneal tomography was performed for both eyes (Pentacam High Resolution, Oculus, Inc, Wetzlar, Germany) and revealed a significant increase in corneal curvature, with corneal irregularity and asymmetry in the axial and posterior maps, and a decrease in corneal thickness (Figure 2).

OD: Right eye. OS: Left Eye.

The patient complained of burning, tearing, itching, and red eye. He received antiglaucoma eye drops (beta-blocker) and corticosteroids in the right eye as a post-corneal transplantation prescription.

**Figure 3:**
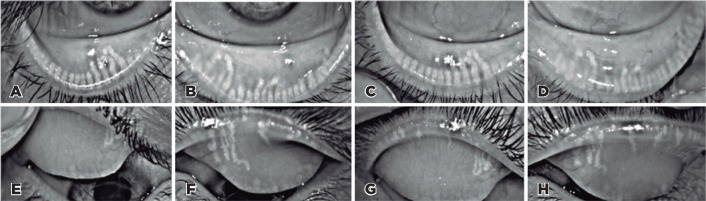
Meibography images. (A) Right lower eyelid before treatment. (B) Left lower eyelid before treatment. (C) Right lower eyelid after treatment. (D) Left lower eyelid after treatment. (E) Right upper eyelid before treatment. (F) Left upper eyelid before treatment. (G) Right upper eyelid after treatment. (H) Left upper eyelid after treatment.

Lower and upper eyelid meibography and tear meniscus height were captured with the Oculus Keratograph 5M (Oculus, Inc). Meibography revealed a severe meibomian gland dropout in both eyes. In the upper eyelid, virtually no glands were found, and in the lower eyelid, >50% loss was observed (Meiboscore grade 4 in the lower eyelid and 2 in the upper eyelid - Figure 3).

**Figure 4:**
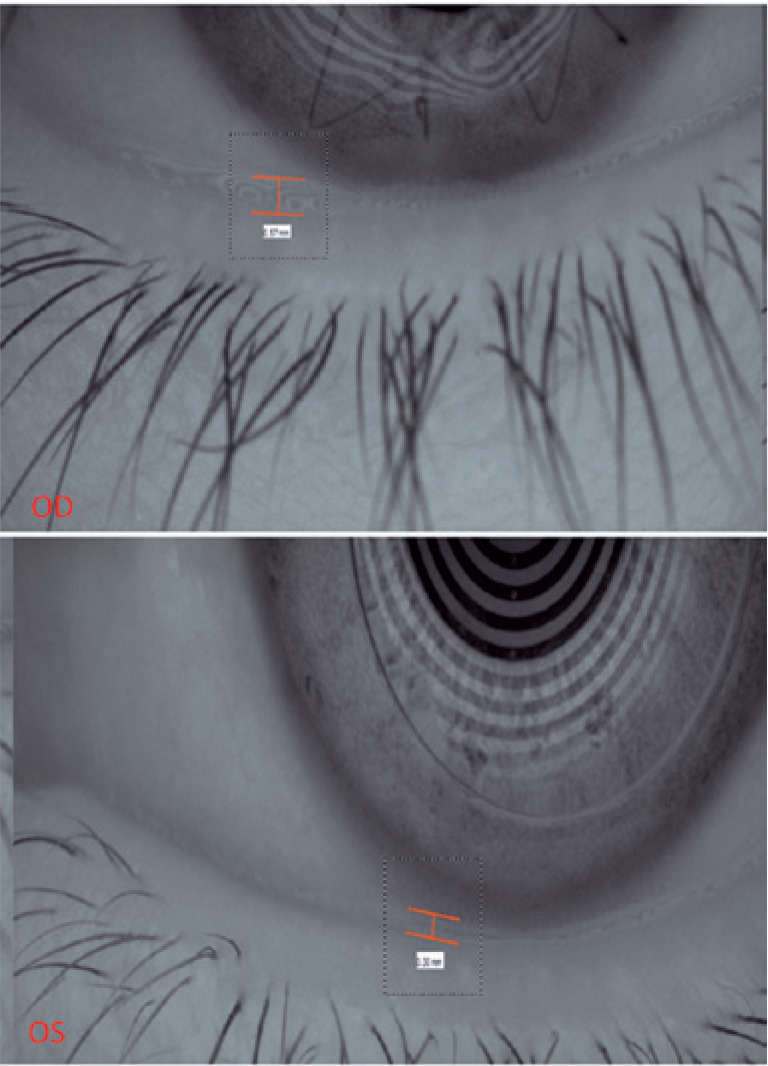
Tear meniscus height.

The tear meniscus height was slightly increased in both eyes, 0.57 mm in the right eye and 0.3 mm in the left eye (normal, >0.2 mm). This finding may represent a compensatory mechanism induced by the increased tear evaporative rate (Figure 4).

OD: Right eye. OS: Left Eye.

On the basis of the findings described earlier, a diagnosis of MGD was made, with consequent evaporative dry eye. Preservative-free lubricant, eyelid hygiene, and massage after warm compresses were prescribed to improve the health and symptoms of the ocular surface. The patient returned after 2 months for evaluation, reporting improvement of symptoms, and the treatment was maintained. On biomicroscopic examination, the MGD findings remained as in the initial examination. On meibography, the meiboscore grades were 3 and 4 in the lower and upper eyelids, respectively (vs 2 and 4, respectively, in the initial evaluation; Figure 3). The tear meniscus heights were 0.50 and 0.39 mm in the right and left eyes, respectively (vs 0.57 and 0.30, respectively, in the initial evaluation).

## DISCUSSION

Chronic blepharitis and MGD are among the most common diseases of the eyelids, which lead to signs and symptoms of dry eye, corneal vascularization, inflammatory infiltrates, and persistent eye rubbing^(6,7)^. As previously exposed, eye rubbing resulting in sheer strength reduction and cone-forming deformation was described as a possible etiological factor of keratoconus^(8)^.

Herein, we describe a case of advanced keratoconus in which the patient had multiple factors for the perpetuation of dry eye and ocular surface inflammation, such as a postoperative condition, rigid CL wearing, and meibomian gland deficiency. No appropriate treatments for the dry eye and MGD were initiated until follow-up. Objective propaedeutic tests such as tear meniscus height and meibography, performed using Keratograph represent important tools for dry eye diagnosis and proper evaluation of the ocular surface and tear film. This case demonstrates the importance of meibomian gland dropout and increased TMH as compensatory mechanisms.

Thus, treatment of underlying chronic inflammations such as dry eye, MGD, and blepharitis might be important to prevent keratoconus progression and guarantee symptom relief. Eye rubbing could contribute to the worsening of blepharitis, partly by transferring pathogens to the eyelids. In view of this, patients should be instructed about eyelid hygiene in addition to refraining from eye rubbing.

Patients with keratoconus who wear RGP CLs often present signs of mechanical trauma to the lens. This, in combination with frequent eye rubbing, typically leads to a strong inflammatory response mediated by cytokine release in the corneal epithelium. This inflammation, in turn, induces apoptosis of the corneal stromal cells and fibroblasts. Thus, this inflammatory circle may trigger perpetuated corneal, ocular surface, and tear film diseases.

Ong and Larke^(9)^ reported that the prevalence of MGD among CL wearers was 30%, which is significantly higher than that (20%) among non-CL wearers. Similarly, Li et al.^(10)^ reported blepharitis and MGD in 31.91% of CL wearers. Many studies have examined the relationship between CL wearing and meibomian gland changes. Such studies have found that lens wearing is associated with adverse changes in the meibomian gland morphology and in the conditions of the lid margin and meibum, suggesting that CL wearing negatively impacts meibomian glands^(11-14)^. CL wearers have a significantly greater degree of meibomian gland loss than non-wearers. This suggests that meibomian gland loss might be one of the underlying mechanisms of CL-related dry eye^(15)^. Paugh et al.^(16)^ showed that the symptoms reported by CL wearers were ameliorated by improvement of lid hygiene and eyelid massage, suggesting that MGD was related to the complaints of the CL wearers.

In the present case, the patient had many distinct factors that contributed to the ocular surface inflammation and epithelial trauma, such as postoperative inflammation of the penetrating right eye transplant and the use of rigid CLs in the left eye. In older patients, the classic signs of atopic conjunctivitis are not always present, and further investigation of the causes of chronic rubbing is necessary, as evaporative dry eye due to significant loss of the meibomian glands was present in our case. At the follow-up visit, the patient reported important improvement of the symptoms, in spite that the biomicroscopy, meibography, and THM examination findings were similar to those in the initial evaluation. This demonstrates that in cases such as our case, the improvement of symptoms may be dissociated from the improvement of the eyelid aspect and complementary examination findings, which take longer to change.

Therefore, detection of meibomian gland deficiency in patients with keratoconus is mandatory. Patients with keratoconus often require multiple surgical and non-surgical interventions. Clinicians must raise awareness among patients that improvement of the eye surface and decreased rubbing are important treatment goals. The appropriate treatment would reduce burning sensation, irritation, tearing, photophobia, blurred vision, and red eyes related to dry eye and ocular surface inflammation, thereby decreasing eye rubbing. Effective management of dry eye disease might decrease eye rubbing and, thus, the mechanical stress on the already vulnerable corneas. This case report demonstrates significant alterations in the ocular surface parameters and meibomian gland morphology in a patient with keratoconus. We hope that these findings inspire future studies about the associations of such relevant ocular conditions, which profoundly impact patient quality of life and vision.
